# Demographic and ecological risk factors for human influenza A virus infections in rural Indonesia

**DOI:** 10.1111/irv.12468

**Published:** 2017-08-09

**Authors:** Elisabeth Dowling Root, Dwi Agustian, Cissy Kartasasmita, Timothy M. Uyeki, Eric A. F. Simões

**Affiliations:** ^1^ The Ohio State University Columbus OH USA; ^2^ Faculty of Medicine Hasan Sadikin General Hospital Universitas Padjadjaran Bandung Indonesia; ^3^ Influenza Division National Center for Immunization and Respiratory Diseases Centers for Disease Control and Prevention (CDC) Atlanta GA USA; ^4^ University of Colorado School of Medicine Aurora CO USA; ^5^ Center for Global Health Colorado School of Public Health Aurora CO USA

**Keywords:** age structure, birds, geographic information system, H1N1pdm09, H3N2, risk factors

## Abstract

**Background:**

Indonesia has the world's highest reported mortality for human infections with highly pathogenic avian influenza (HPAI) A(H5N1) virus. Indonesia is an agriculturally driven country where human‐animal mixing is common and provides a unique environment for zoonotic influenza A virus transmission.

**Objectives:**

To identify potential demographic and ecological risk factors for human infection with seasonal influenza A viruses in rural Indonesia, a population‐based study was conducted in Cileunyi and Soreang subdistricts near Bandung in western Java from 2008 to 2011.

**Methods:**

Passive influenza surveillance with RT‐PCR confirmation of influenza A viral RNA in respiratory specimens was utilized for case ascertainment. A population census and mapping were utilized for population data collection. The presence of influenza A(H3N2) and A(H1N1)pdm09 virus infections in a household was modeled using Generalized Estimating Equations.

**Results:**

Each additional child aged <5 years in a household increased the odds of H3N2 approximately 5 times (OR=4.59, 95%CI: 3.30‐6.24) and H1N1pdm09 by 3.5 times (OR=3.53, 95%CI: 2.51‐4.96). In addition, the presence of 16‐30 birds in the house was associated with an increased odds of H3N2 (OR=5.08, 95%CI: 2.00‐12.92) and H1N1pdm09 (OR=12.51 95%CI: 6.23‐25.13).

**Conclusion:**

Our findings suggest an increase in influenza A virus infections in rural Indonesian households with young children and poultry.

## 1 INTRODUCTION

Influenza pandemics and seasonal influenza epidemics have caused high mortality and morbidity with devastating global economic losses.^1^, ^2^ Between 2003 and 2015, Indonesia, Vietnam, and Egypt reported the highest number of human highly pathogenic avian influenza (HPAI) A(H5N1) cases. As of 2015, Indonesia has had the greatest number of deaths, with fatalities occurring yearly since 2005.^3^ Tropical regions such as Indonesia are a suspected source of antigenically drifted seasonal influenza A(H3N2) virus strains which may migrate to the Southern Hemisphere and Northern Hemisphere through the global air traffic network.^4^ Indonesia, a largely agricultural country where human‐animal mixing is common, provides a unique environment for zoonotic influenza A virus transmission and an ideal ecological setting for the generation of novel influenza A viruses.^5^ A significant amount of research has focused on understanding the risk factors associated with HPAI H5N1 viral infection. Studies from Indonesia and elsewhere show that the majority of human infections with HPAI H5N1 virus have been associated directly or indirectly with poultry exposure including close contact with sick or dead poultry, visiting a live poultry market and commercial poultry density.^6^, ^7^, ^8^, ^9^, ^10^ A recent review of the literature concluded that direct exposure to birds was one of the most likely sources of human infection with A(H5N1).^11^ Other studies investigating area‐level ecological correlates of HPAI suggest that rice paddy fields, population density, and exposure to potentially contaminated water sources 6, 7, 12, 13, 14 all increase risk of infection.

While human infection with HPAI A(H5N1) virus in Indonesia has occurred sporadically, seasonal influenza A and B viruses regularly circulate within the population. Several influenza surveillance studies have been conducted in Indonesia which suggest that the burden of seasonal influenza is high and that influenza viruses appear to circulate year‐round with increased activity between November and March.^15^, ^16^, ^17^, ^18^ These studies have focused on determining the burden of disease, characterizing circulating virus strains, and understanding seasonal trends. We are not aware of any studies of seasonal influenza in Indonesia that have examined risk factors beyond age and sex. In fact, much of what we know regarding the risk factors of community‐acquired seasonal influenza is derived from research conducted in middle‐ and high‐income countries. These studies reinforce that factors such as the number of school age children, household and population age structure,^19^, ^20^, ^21^ contact patterns,^22^, ^23^ and interaction with birds or bird environments 24, 25, 26 are associated with influenza distribution in a community.

The intent of this study was to explore the role of the animal‐human interface in community‐acquired seasonal influenza. This is a population‐based exploration of ecological risk factors driving symptomatic influenza A virus infections defined by care seeking at first‐level health facilities. We combined passive influenza surveillance with a complete population census to determine the influence of household demographic characteristics, birds in the household, and community‐level population structure on the risk for symptomatic influenza A virus infections.

## 2 METHODS

### 2.1 Study area

**Figure 1:**
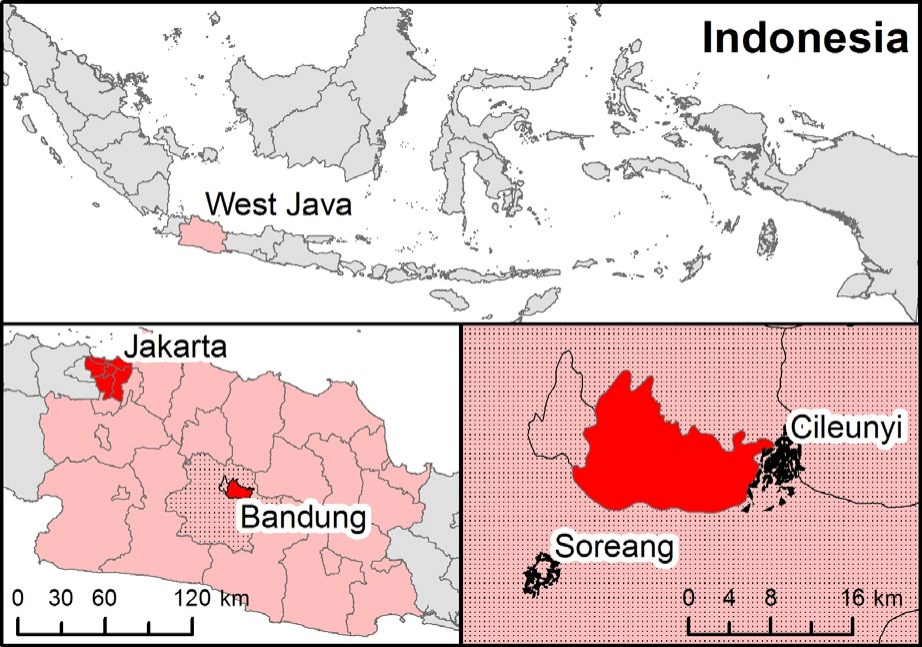
Location of the study area in Bandung District, West Java Province, Indonesia.Map shows the location of Soreang and Cileunyi relative to other major Indonesian cities. The two subdistricts are located in West Java province just outside the city of Bandung. Bandung is Indonesia's third largest city located approximately 140 km southeast of Jakarta

This study was conducted in Cileunyi and Soreang subdistricts in rural West Java Province, approximately 18 km outside the provincial capital of Bandung city. A map of the study area is shown in Figure 1. Cileunyi and Soreang lie in a river basin 768 m above sea level, surrounded by volcanic mountains with a salubrious climate, with an annual average temperature of 23.6**°**C and 1000‐3500 mm of rain. The 2010 population was 104 696 in Cileunyi and 61 211 in Soreang, in a combined area of about 21 km^2^. Both government and private clinics provide primary health care, and there are three government community health centers that serve the area.

### 2.2 Study population and data

This study used a prospective cohort design to identify ILI cases in Soreang and Cileunyi. Patients with symptomatic influenza virus infections were identified by passive surveillance in three government community health centers (puskesmas) in the study area between October 2008 and September 2011. Dedicated, trained study physicians screened and enrolled all patients visiting the clinics with signs and symptoms of influenza‐like illness (ILI). To be included in the study, participants must have: (i) lived in the study area at the time of illness, (ii) presented with ILI defined as a fever (body temperature >37.5°C) with cough or sore throat, and (iii) had signed informed consent. Basic demographic data, address, and clinical information of all eligible subjects were recorded, and nasal and oropharyngeal swabs were collected for influenza virus testing at Hasan Sadikin General Hospital Laboratory. Approximately 2 weeks after enrollment, trained nurses conducted home visits and administered a survey to collect information on clinical outcomes and household and potential environmental risk factors. The geographic location of each participant's household of residence was collected using a handheld GPS receiver and linked to study data using a Geographic Information System (GIS). If a participant presented to the puskesmas with an ILI more than once during the study period, but at least 14 days apart, each visit was treated as a separate event. The patient was screened again, and a second nasal and oropharyngeal swab was obtained. If the patient was identified as having influenza from laboratory results, this was recorded as a separate case from the first and recorded as a second case in the household (ie, a household could have two separate records if both H3N2 and H1N1pdm09 viruses were identified during these two visits).

To collect data on the total population at risk, 448 trained local community health workers (CHWs) conducted a census of all households in the two study subdistricts. The CHWs used standardized forms, which collected data on: address of residence, the age, sex and education of all permanent household residents, and number of birds and poultry kept by the household. Twelve dedicated trained field surveyors conducted quality control to identify missing values and errors of transcription on the forms. They subsequently conferred with the CHWs to correct the data entry forms. Following the creation of the population list and address validation, the 12 field surveyors conducted door‐to‐door visits to geocode the household using handheld GPS receivers. This mapping activity was used as a secondary quality control check to validate the population data initially collected by the CHWs.

### 2.3 Laboratory testing

Nasopharyngeal (children and adults) or oropharyngeal (adults) swab samples were obtained from each enrolled patient and transported at 4‐8**°**C to the Research Laboratory in a Universal Virus transport medium (Becton‐Dickinson, Franklin Lakes, NJ, USA).

Influenza A and B viral RNA was detected in a one‐step multiplex real‐time RT‐PCR using primers and specific LNA‐mediated TaqMan probes in two separate assays, using standard protocols.^27^ Briefly, the first assay consisted of primers and probes specific to the matrix (M1) gene of influenza A virus, influenza B virus, and host glyceraldehyde‐3‐phosphate dehydrogenase (GAPDH) gene. The second multiplex assay detected influenza A virus subtypes using primers and probes (1st Base, Singapore) specific for regions of the H1, H3, and H5 hemagglutinin (HA) genes. The probes were labeled with three different fluorescent reporter dyes (FAM, HEX, and Cy5 with emission wavelengths at 518, 556, and 667 nm, respectively). Specimens, which tested positive for influenza type A, were subtyped in a second real‐time RT‐PCR assay incorporating primers and probe specific for H1N1pdm09 using the standard CDC protocol.^28^ Due to resource limitations, further characterization of the non‐subtypeable specimens was not performed for this study.

### 2.4 Household covariates

Using the population census in the GIS, we developed a number of demographic and ecological variables at the neighborhood and household levels that were examined for a relationship with influenza A cases. We focused on influenza A cases as our primary goal was to identify ecological risk factors at the human‐animal interface. For each household, we calculated the average household size, whether the head of household had less than a high school education, the age structure of the household, the distance from each household to the nearest puskesmas and several indicators of the number of birds kept by the household (presence/absence and total number). Households were also grouped into “neighborhoods”—one of 224 subcommunities within the subdistricts. Using a Geographic Information System (GIS), neighborhood‐level variables were created by drawing a 200‐m radius buffer around each household and aggregating data within the buffer. For each household neighborhood, we calculated the population density, percent of households where the head of household had less that a high school education, percent of the population in one of five age categories (0‐5, 6‐15, 16‐50, 51‐65 and >65 years) and the total number of birds within a 200‐m radius of each household. To control for potential bias due to underreporting, we created a measure of the neighborhood healthcare utilization and calculated as the percentage of households that used community healthcare services for ILI during the study period.

### 2.5 Statistical approach

The occurrence of a subject with symptomatic influenza A virus infection seeking care at the puskesmas in each household was modeled using generalized estimating equations (GEE) with the logit link function. The GEE models used correlation matrices that were independent and exchangeable within neighborhoods. The use of exchangeable neighborhood matrices corrects for potential spatial correlation in the data due to local‐level transmission dynamics of influenza viruses. GEE was implemented by R version 2.15.2 with the *geepack* library.

Each of the two different influenza A subtype virus infections (H3N2 and H1N1pdm09) were modeled separately. In the models, the occurrence (yes/no) of an influenza A case in each household was the primary outcome variable. Covariates with a known association with influenza virus infection were included to control for potential confounding. The full set of covariates we considered can be found in Table 1; the set of covariates and their specification used in the final model are shown in Table 2. This final model was chosen based on minimization of QIC (a measure similar to AIC for GEE models which show relative goodness of fit across model specifications) and through an analysis of the area under the receiver operating curve (AROC; which shows how accurately models predict the outcome). Higher values of AROC (>0.80) indicate the model is “good” at predicting household influenza cases.

**Table 1:** Descriptive statistics of sample households, Cileunyi and Soreang subdistricts, Indonesia, October 2008‐September 2011

Variable	Controls^a^	Cases	
	(n=42 408)	H3N2 (n=171)	H1N1pdm09 (n=149)
	% Mean (SD)	% Mean (SD)	% Mean (SD)
Household‐level Variables			
Household head less than high school education (%)	54.4	60.6	51.7
Average household size (#)	3.86 (1.46)	5.00 (1.84)	4.80 (1.63)
Male (%)	49.6 (19.8)	51.0 (17.3)	51.9 (17.9)
Family age structure (# per household)			
0‐5 y	0.20 (0.42)	0.72 (0.75)	0.55 (0.61)
6‐15 y	0.80 (0.82)	1.15 (0.89)	1.34 (0.93)
16‐50 y	2.30 (1.10)	2.71 (1.20)	2.55 (1.17)
51‐65 y	0.40 (0.67)	0.35 (0.65)	0.31 (0.62)
>65 y	0.15 (0.44)	0.08 (0.33)	0.05 (0.25)
Birds kept in the household (% of households)			
No birds	83.42	68.42	64.43
1‐5 birds	11.23	12.28	18.12
6‐15 birds	4.09	9.94	8.05
16‐30 birds	0.94	2.92	6.04
>30 birds	0.32	6.43	3.36
Distance to healthcare facilities (km)	3.19 (1.91)	2.71 (1.11)	2.72 (1.15)
Neighborhood‐level Variables (within 200 m/radius)			
Household head less than high school education (%)	54.83 (25.46)	59.73 (18.67)	55.02 (19.77)
Population density (people per m^2^)	0.96 (0.01)	0.80 (0.03)	0.94 (0.04)
Bird density (birds per m^2^)	0.30 (0.56)	0.25 (0.37)	0.35 (0.41)
Community healthcare utilization rate (%)	5.91 (6.63)	12.89 (11.07)	13.39 (11.02)

a The number of controls was calculated by subtracting the number of households with a laboratory‐confirmed influenza case at any time during the study from the total households recorded in the population census.

**Table 2:** Odds ratios and 95% confidence intervals bivariate and multivariable models of the risk of household influenza a virus infection among 42 775 households in Cileunyi and Soreang subdistricts, Indonesia, October 2008‐September 2011

Variable	H3N2				H1N1pdm09			
	OR^a^	95% CI	AOR^b^	95% CI	OR^a^	95% CI	AOR^b^	95% CI
Household variables								
Household head less than high school education	1.29	(0.95‐1.75)	1.16	(0.78‐1.73)	0.89	(0.65‐1.23)	0.91	(0.55‐1.52)
Total household size (#)	1.44	(1.35‐1.55)	**1.13**	**(1.00‐1.27)**	1.38	(1.27‐1.48)	1.00	(0.85‐1.17)
Family age structure (# per household)								
0‐5 y	4.99	(4.02‐6.19)	**4.59**	**(3.30‐6.24)**	3.50	(2.72‐4.50)	**3.53**	**(2.51‐4.96)**
6‐15 y	1.57	(1.34‐1.84)	**1.31**	**(1.06‐1.62)**	1.87	(1.60‐2.19)	**1.77**	**(1.35‐2.34)**
51‐65 y	0.88	(0.69‐1.13)	0.99	(0.74‐1.34)	0.80	(0.62‐1.05)	1.06	(0.77‐1.45)
>65 y	0.56	(0.34‐0.92)	0.65	(0.38‐1.11)	0.41	(0.22‐0.79)	**0.53**	**(0.29‐0.96)**
Birds kept by the household								
0 birds	Ref		Ref		Ref		Ref	
1‐5 birds	1.35	(0.84‐2.14)	1.51	(0.83‐2.77)	2.09	(1.36‐3.21)	**2.65**	**(1.56‐4.50)**
6‐15 birds	3.00	(1.80‐4.99)	**3.64**	**(1.97‐6.69)**	2.56	(1.40‐4.67)	**3.58**	**(1.89‐6.78)**
16‐30 birds	3.80	(1.54‐9.36)	**5.08**	**(2.00‐12.92)**	8.27	(4.15‐16.49)	**12.51**	**(6.23‐25.13)**
>30 birds	24.78	(13.05‐47.05)	**30.90**	**(14.40‐66.32)**	13.60	(5.45‐33.97)	**21.23**	**(6.90‐65.31)**
Distance to health center (km)	0.86	(0.79‐0.94)	**0.81**	**(0.73‐0.91)**	0.87	(0.79‐0.95)	**0.87**	**(0.78‐0.98)**
Neighborhood variables (within 200 m/radius)								
Household head less than high school education (%)	1.00	(0.99‐1.01)	0.99	(0.99‐1.01)	0.99	(0.98‐1.00)	0.99	(0.98‐1.00)
Population density	0.93	(0.91‐0.97)	**0.93**	**(0.89‐0.98)**	1.01	(0.97‐1.05)	0.99	(0.98‐1.03)
Bird density	0.68	(0.33‐1.42)	0.79	(0.42‐1.47)	1.23	(0.93‐1.37)	1.18	(0.94‐1.50)
Community healthcare utilization rate (%)	1.08	(1.06‐1.09)	**1.07**	**(1.06‐1.09)**	1.08	(1.07‐1.09)	**1.08**	**(1.07‐1.10)**
AROC^c^			0.83				0.84	

Note: Bolded values indicate statistically significant (*P*<.05) values.

a OR=Crude odds ratios from bivariate models.

b AOR=Adjusted odds ratios from multivariate models.

c AROC=Area under the receiver operating curve (ROC).

### 2.6 Ethical considerations

This study was approved by the Health Research Ethics Committee, Faculty of Medicine, University of Padjadjaran (Approval #07/UN6.C2.1/KEPK/PN/2012), COMIRB of the University of Colorado, Denver, and the Centers for Disease Control Atlanta.

## 3 RESULTS

The population census recorded 163 014 individuals in 42 775 households, for an average of 3.8 people per household. Of the 3356 enrolled ILI subjects, 402 had influenza A and 105 had influenza B. Of the former, 193 were H3N2, 157 were H1N1pdm09, 9 were seasonal H1N1, and 43 were not subtypeable. On follow‐up 2 weeks later, none of these subjects were hospitalized or died. The multivariable statistical analysis modeled household‐level risk, so the 193 H3N2 cases corresponded to 171 unique households, the 157 H1N1pdm09 cases corresponded to 149 households, the 43 non‐subtypeable were found in 38 households, and the 9 seasonal H1N1 were in 9 households. These sample sizes are reflected in Table 1. The analysis presented here focuses on households with influenza A as we had a sample size suitable for multivariable analysis and our primary goal was to study the animal‐human interface. Furthermore, resource limitations did not allow for characterization of the non‐subtypeable specimens, so we also excluded these in multivariable modeling.

Table 1 shows the characteristics of the households in the sample. Households with H3N2 or H1N1pdm09 cases were slightly larger and had a larger population of children <15 years and a greater number of birds kept by the house. These households also had a much lower healthcare utilization rate. Bivariate associations between the presence of an influenza A case (yes/no) in a household and ecological factors of interest are presented in Table 2. At the household level, average household size, the number of children aged <5 years, and presence of birds/poultry were significantly associated with the presence of H3N2 and H1N1pdm09 cases. At the neighborhood level, population density and neighborhood age structure were significantly associated with lower odds of H3N2 virus infections only, while neighborhood community healthcare utilization was significantly associated with both subtypes.

**Figure 2:**
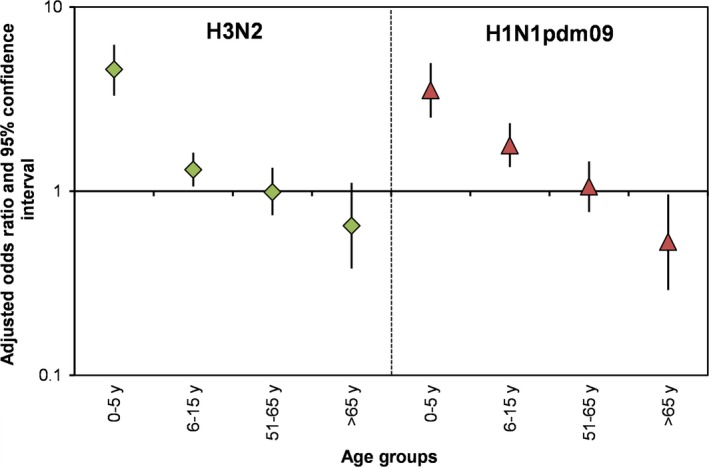
Plot of odds ratios and 95% confidence intervals from multivariable models for risk of household influenza a virus infection by subtype and age group

**Figure 3:**
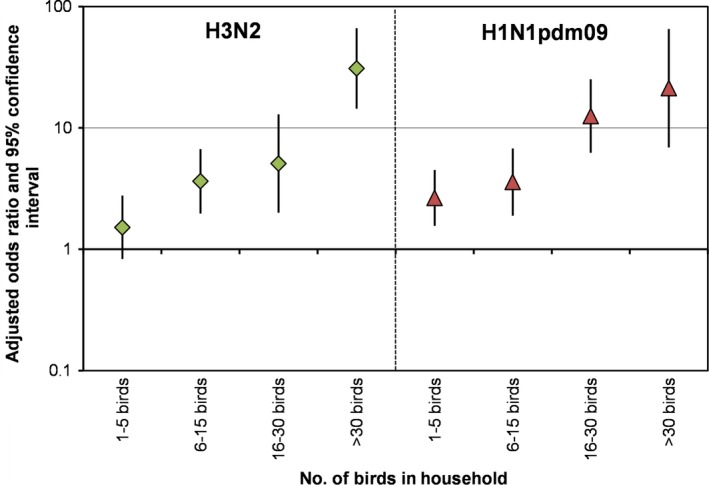
Plot of odds ratios and 95% confidence intervals from multivariable models for risk of household influenza a virus infection by subtype and number of birds kept by the household

In the multivariable analyses, household size, number of children aged <6 years and 6‐15 years, and presence of birds/poultry in the household were consistently and significantly associated with an increased odds of H3N2 and H1N1pdm09. Larger households had an increased odds of H3N2 (OR=1.134, 95%CI: 1.00‐1.27) but not for H1N1pdm09 (OR=1.008, 95%CI: 0.85‐1.17) cases. Each additional child aged <5 years in a household increased the odds of H3N2 cases in that household 4.5‐fold (OR=4.59, 95%CI: 3.30‐6.24) and H1N1pdm09 cases 3.5‐fold (OR=3.53, 95% CI: 2.51‐4.96) (Figure 2). We also noticed a decrease in the odds of H1N1pmd09 in households with a larger number of >65 year olds (OR=0.29, 95% CI: 0.29‐0.96). Households that kept 6‐15 birds had an increased odds of H3N2 (OR=3.64, 95%CI: 1.97‐6.69) and H1N1pdm09 (OR=3.58, 95%CI: 1.89‐6.78) when compared to households with no birds (Figure 3). Odds of H3N2 and H1N1pmd09 increased as the number of birds kept by the household increased (eg, 16‐30 and >30 birds). We note that the confidence intervals for the highest two categories (16‐30 and >30 birds) are quite large due to small sample sizes. At the neighborhood level, greater population density was associated with a decreased risk for H3N2. The only neighborhood variable significantly related to both H3N2 and H1N1pdm09 influenza A cases (after controlling for household characteristics) was community healthcare utilization. The odds of influenza A virus infection (for both subtypes tested) was significantly higher in communities with high healthcare utilization.

## 4 DISCUSSION

We found an increased risk of influenza A cases in households with children aged <6 years and 6‐15 years that was consistent across the categories of influenza A virus subtypes. Elevated risk of influenza A virus infection was also observed for households with a larger number of older children (6‐15 years), although the effect was smaller than that observed with younger age groups. This age group effect appeared to decrease with age, such that larger numbers of older individuals (>65 years) conferred a protective effect for the household, although this relationship was only significant for H1N1pmd09. In general, this result is consistent with other studies that examined individual‐level influenza risk factors.^19^, ^29^, ^30^ A study in Tampa Bay, Florida, found that the seroprevalence of antibodies to H1N1pdm09 virus decreased by age, from the highest frequency of 53% in children aged 5‐17 years, to the lowest proportion of 11%‐13% in adults aged >50 years. This study also showed decreased susceptibility with increases in age.^31^ A longitudinal community‐based cohort study in Hong Kong also showed that increased age conferred a protective effect for H1N1pdm09 virus infections.^30^ A community‐based cohort study in Vietnam in 2007‐2010 also showed that individuals aged 20 years or less had higher risk of H3N2 and H1N1pdm09 virus infection compared to those aged >20 years.

As this is an ecological study at the household level, there was no information regarding which family member had influenza A virus infection. Although households with young children experienced an increased risk of infection, this does not necessarily mean that a young child had influenza A. However, evidence from community cohort studies that assessed individual‐level data showed that young children are the most susceptible to influenza among family members.^32^ It is biologically plausible that family age structure as an ecological risk factor might simply represent the age‐specific risk of each family member for influenza A.

The number of school age children (6‐15 years old) also had a significant effect on H3N2 and H1N1pdm09 virus infections in households. This finding is supported by the existing literature, which suggests that school age children are likely to introduce influenza A virus to the household.^33^ Therefore, the association of family age structure with influenza A in the household is likely not solely due to the age‐related immune response or susceptibility, but may also be associated with age‐related contact pattern heterogeneity, such as interaction with other persons between school or workplace. Unfortunately, we did not collect contact or activity pattern information to explore these dynamics further.

The number of birds or poultry in the household was also associated with symptomatic influenza A virus cases. This association was relatively weak with the ownership of 1‐5 birds, but became stronger with an increasing number of birds in the household. Figure 3 illustrates this dose‐response relationship. This trend was consistently found across both influenza A subtype outcomes. While it is biologically implausible that there is a causal link of seasonal influenza A virus infection of humans to the presence of birds, there could be a potential environmental/immunological explanation for our observations. A larger number of poultry kept by rural households would be associated with more fecal and hence bacterial environmental contamination.^34^, ^35^ Bacterial lipopolysaccharide (LPS) has been shown to protect mice against HPAI virus infection through toll‐like receptor stimulation^36^ and might explain the relative mildness of the illness seen in our rural population, as none of them were subsequently hospitalized or died. Bacterial LPS has also been shown to inhibit the induction of CD8+ T‐cell immunity by influenza virus infection.^37^ One possibility is that high levels of bacterial LPS in the environment, by suppressing T‐cell responses, increase the ratio of asymptomatic to symptomatic influenza virus infections, leading to our observed higher rates. Our observation that the relationship is dose‐dependent supports this hypothesis.

Another potential explanation for this finding is related to sanitation and associated health behaviors or household socioeconomic status. Handwashing and hand hygiene have been highly publicized as a core management strategy for influenza and other respiratory disease. Although handwashing is effective in reducing the incidence of common diseases such as acute respiratory infections, data on its effectiveness specifically for community‐acquired virus influenza infections are limited. A number of randomized controlled trials have found little or no evidence of a reduction in laboratory‐confirmed seasonal influenza with proper hand hygiene,^38^, ^39^, ^40^, ^41^ although a more limited set of studies show a modest reduction in risk.^42^, ^43^ This study did not collect data on health behaviors, so it is possible that households with a reduced risk of influenza also had better hand hygiene practices. Related to this, households with poor or no access to clean water for washing may also have poorer hand hygiene or may use contaminated water sources. Lack of an indoor water source has been shown to increase risk of avian influenza^10^ although the mechanisms for this relationship are not clear. Likewise, low socioeconomic status is often indicative of a lack of access to sanitation/clean water and less desirable health behaviors. The population census did not collect information on access to clean water and sanitation or on household socioeconomic status, so we cannot test this relationship with our data.

Whatever the mechanism for the association between infection and birds kept by the household, it is clear that human‐poultry interactions are important for avian‐to‐human transmission of HPAI H5N1 virus in Indonesia. This is a common interaction in both urban and rural areas in Indonesia.^44^, ^45^, ^46^, ^47^ Poultry in the home also increases the possibility that reassortment of avian and human influenza A virus strains might occur in humans in these households.^48^

Another interesting finding was the lack of neighborhood‐level associations between influenza A cases and population density and bird density. Once we controlled for household‐level risk factors, these neighborhood‐level factors (which were significant in bivariate models) were no longer significantly associated with risk of influenza A virus infection. The one exception was population density for H3N2 virus infection, where increased density conferred a slight protective effect. This result was surprising as higher population density would likely facilitate increased influenza virus transmission. Areas with higher population density have fewer birds kept by the household, which may correspond to the decreased risk in these areas. The fact that age structure is associated with the outcomes at the household scale and not at the neighborhood level reflects two potential limitations of our analysis. First, while a household's neighborhood (defined by a 200‐m buffer around each household) is practical and captures daily interactions with neighbors in this rural setting, this neighborhood size might not adequately capture a larger spatial transmission processes, which could very well occur beyond the 200‐m radius. Second, this finding has led us to hypothesize that social group contacts, such as at school and the workplace (which exist beyond the local neighborhood), may play a more important role in influenza A virus transmission as compared to local geographically based contacts. Unfortunately, we do not have data on the school and/or workplace‐based contacts patterns. This will be an important subject for further investigation.

Several limitations should be noted. Cases were collected using a passive surveillance study in a community healthcare center, and therefore, the influenza A virus infections identified in this study represent only those populations who utilized the facilities. There are two potential limitations of this approach. First, research suggests that many seasonal influenza virus infections are believed to be asymptomatic, with some proportion of infection resulting in mild acute respiratory illness without fever.^49^ The passive surveillance strategy we employ here relies on symptomatic patients who seek health care at one of the puskesmas study sites, which underestimates the number of influenza virus infections that have occurred in a household. Passive surveillance also leads to detection bias among households that are geographically more distant from the puskesmas because people are less likely to seek care. We attempted to control for this bias by including a measure of neighborhood health service utilization in the multivariable models. Second, the characteristics of people who chose to utilize healthcare facilities may differ from those who do not as utilization is a complex and multidimensional concept. It is therefore likely that some unmeasured confounding exists. We attempted to adjust for this by controlling for community‐level utilization of health care. Multivariable models across all outcomes showed that the neighborhood healthcare utilization showed a significant association with the number of influenza A virus infections in the household, indicating this variable controls for households with no reported infections to some degree. Finally, our field surveyors who conducted door‐to‐door validation on bird ownership were blinded to the outcome result, and therefore, we believe any systematic exposure misclassification is minimal. We assume any bias occurred randomly and therefore would be more likely to bias the result toward the null.

Some migration and mobility are inevitable over the course of the 3 years of the study. We note that the location of the patient at the time of diagnosis is accurate, as a nurse visited their home 2 weeks after the community health center visit and collected household information. However, household data on the “control” population in the study—all households with no reported influenza case—were only collected once at the during the initial population census. Some changes to household structure could certainly happen during the study period, perhaps leading to a misrepresentation of the control population. However, we note that there is little migration out of this study area, and people do not typically move residences as most households are engaged in agriculture and more or less tied to their land. Thus, we believe these biases are minimal.

## 5 CONCLUSION

We found that age structure and the number of birds in the household were significantly associated with influenza A virus infections and these ecological determinants operate at the household level rather at the neighborhood level. In this exploratory study, the positive association between household birds and seasonal influenza A virus infections suggests an underlying common ecological factor, as yet unidentified. Regardless of the mechanism, in these populations where avian influenza A viruses may be prevalent among poultry, the possibility of reassortment of HPAI H5N1 virus or other avian influenza A viruses with seasonal influenza A viruses is a theoretical possibility. Our results suggests that further studies examining the relationship between human behavior, animal exposure, and influenza A virus evolution and novel influenza A virus emergence are indicated in rural Indonesia.

## DISCLAIMER

The findings and conclusions in this report are those of the authors and do not necessarily represent the official position of the Centers for Disease Control and Prevention.
